# One reference genome is not enough

**DOI:** 10.1186/s13059-019-1717-0

**Published:** 2019-05-24

**Authors:** Xiaofei Yang, Wan-Ping Lee, Kai Ye, Charles Lee

**Affiliations:** 1 Department of Computer Science and Technology, School of Electronic and Information Engineering, Xi’an Jiaotong University, Xi’an, 710049 China; 2 MOE Key Lab for Intelligent Networks & Networks Security, School of Electronics and Information Engineering, Xi’an Jiaotong University, Xi’an, 710049 China; 3 The Jackson Laboratory for Genomic Medicine, Farmington, CT 06032 USA; 4 Precision Medicine Center, The First Affiliated Hospital of Xi’an Jiaotong University, Xi’an, 710061 China; 5 Genome Institute, First Affiliated Hospital of Xi’an Jiaotong University, Xi’an, 710061 China; 6 Department of Life Sciences, Ewha Womans University, Seoul, 03760 South Korea

## Abstract

A recent study on human structural variation indicates insufficiencies and errors in the human reference genome, GRCh38, and argues for the construction of a human pan-genome.

## Introduction

The human reference genome is a critical foundation for human genetics and biomedical research. The current human reference genome, GRCh38, blends genomic segments from a few individuals, although clones of a single individual predominate [1]. This invites criticisms of the ability of such a reference genome to present the common variants from multiple human populations accurately. In addition, the current human reference genome harbors many genomic segments that actually contain rare variants, and these impact downstream sequence analyses including read alignments and the identification of variants, especially the identification of structural variants (SVs) (that is, insertions, deletions and rearrangements) that encompass more than 50 bp of DNA. Incorporating SVs that are shared among major human populations into the current reference genome can correct for biases and improves both read alignments and the detection of variants in other individuals. Recently, a study based on deep (i.e., > 50×) long-read PacBio whole genome sequencing (WGS) data for 15 individuals from five populations led to the discovery and sequencing of a large fraction of common structural variation. These data can be used to genotype variants from other short-read sequencing datasets and ultimately to reduce biases inherent in the GRCh38 version of the human reference genome [2].

### SV discovery based on long-read sequencing data

**Fig. 1:**
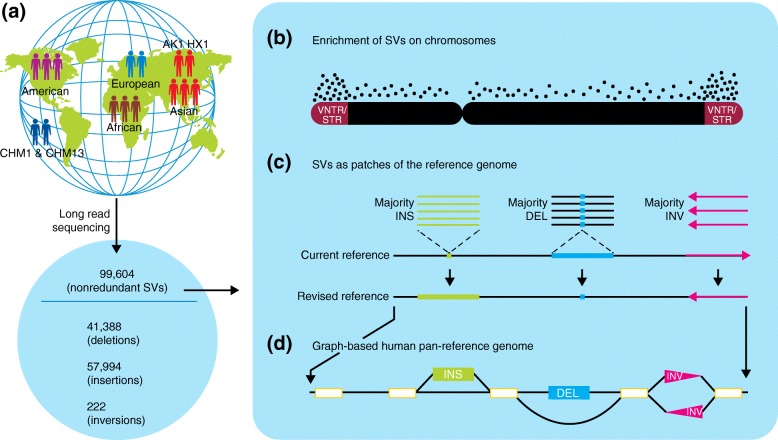
The human genome structural variant (SV) resource. **a** The detection of 99,604 nonredundant SVs in 15 samples from five populations using a long-read sequencing technology. AK1 [4] and HX1 [5] are Asian individuals whose genomes were previously sequenced. **b** The subtelomeric regions of human chromosomes are particularly enriched for SVs of the variable number of tandem repeats (VNTR) and short tandem repeat (STR) types. Here, the frequency of black dots along the length of the chromosome indicates the relative density of SVs. **c** About 15% of the discovered SVs can be found in more than 50% of the samples studied, indicating that these sites actually harbor minor alleles or errors in the current reference genome. **d** Ultimately, a human pan-reference genome can be developed using genome graphs (or other methods) to represent common SVs accurately. *DEL* deletion, *INS* insertion, *INV* inversion

Audano et al. [2] sequenced 11 genomes (from three African, three Asian, two European and three American samples) using single-molecule, real-time (SMRT) PacBio RSII and Sequel long-read sequencing technology. They further analyzed long-read sequencing data, including data from four additional sources: CHM1 [3], CHM13 [3], AK1 [4] and HX1 [5]. Reads were aligned against the GRCh38 version of the human reference sequence using the BLASR software and SVs were detected using the SMRT-SV algorithm [6]. In total, 99,604 nonredundant SVs were identified from these 15 sequenced genomes. The analysis focused on around 95% of the human genome but excluded the pericentromeric and other regions of the genome that are enriched for repetitive DNAs (Fig. 1a). Among the 99,604 discovered SVs, the existence of 2238 ‘shared type’ SVs (shared across all samples) and 13,053 ‘majority type’ SVs (present in more than half of the genomes studied, but not in all samples) suggested that the current reference genome either carries a minor allele or contains an error at each of these positions. These shared and majority SVs were enriched with repetitive sequences and reflect insertions (61. 6 %), deletions (38.1%) and inversions (0.33%). Excluding analyses of the highly repetitive regions of the human genome (which probably contain many SVs), a logarithmic function conservatively suggested that adding SV data from an additional human genome would probably increase the total SV callset by 2.1%, adding 35 genomes would increase the total SV callset by 39% and, finally, adding 327 genomes would identify twice as many SVs than were identified from these 15 genomes.

Among the SVs discovered, 40.8% are novel when compared to previously described SVs from several published large-scale projects (Figure S1E in [2]). To assess the allele frequency of the discovered SVs, Audano et al. [2] went on to genotype these SVs across a total of 440 additional genomes, which were all sequenced using short-read technologies, including those of 174 individuals from the 1000 Genomes Project and 266 individuals from the Simons Genome Diversity Project [7]. The results showed that 92. 6% of the released SVs actually appeared in more than half of the samples, further confirming these biases in the GRCh38 version of the human reference genome.

### SVs enriched with tandem repeat sequences

Audano et al. [2] found that SVs are not randomly distributed across the genome, and in fact, there was as much as a nine-fold increase in SV density within the subtelomeric regions (the last 5 Mb) of human chromosomes. In addition, SVs in these subtelomeric regions were significantly enriched with tandem repeats, particularly for VNTRs (variable number of tandem repeats) and STRs (short tandem repeats), rather than retrotransposons (Fig. 1b). There was also a positive correlation between the abundance of STRs (R = 0.27) and VNTRs (particularly larger VNTRs; R = 0.48) with known hotspots of meiotic double strand breaks (DSBs), suggesting a potential role for DSBs in the formation of SVs in these genomic regions.

### SVs affect gene structures and regulatory elements

How do the discovered SVs interfere with gene expression? To address this question, Audano et al. [2] annotated the shared and majority SVs using RefSeq. The analysis showed that 7550 of these SVs intersect with gene regions (including coding regions, untranslated regions (UTRs), introns, and 2-kb flanking regions), and 1033 of these SVs intersect with known regulatory elements. Some of the SVs disrupted gene structures: 841 intersected RefSeq-annotated coding regions and 667 intersected RefSeq-annotated noncoding RNA regions. For example, a 1.6-kb insertion was located in the 5′ UTR of *UBEQ2L1* and extended into its promoter. In another case, a 1.06-kbp GC-rich insertion was located at the 3′ UTR of *ADARB1* and incorporated motifs that may promote the formation of a quadruplex structure. Examples of SVs located in gene regulatory elements included a 1.2-kb and a 1.4-kb fragment inserted upstream of *KDM6B* and *FGFR1OP*, respectively. These insertions intersected with H3K4Me3 and H3K27Ac sites. Audano et al. [2] further investigated the impact of SVs on gene expression using RNA-seq data from 376 European cell lines and found that the expression of 411 genes was significantly associated with the discovered SVs.

### The discovered SVs can be helpful for re-constructing a canonical human reference genome

GRCh38 currently contains 819 gaps, including minor alleles or actual errors. Audano et al. [2] proposed that the SVs discovered in their work could be included to correct the reference genome (Fig. 1c). They found 34 shared insertions that intersect with scaffold switch-points of the GRCh38 version of the reference genome and the new data could be used to correct possible misassemblies in GRCh38. For instance, a 2159-bp shared insertion overlaps with a switch-point in the *NUTM1* gene and indicates a misassembly by stitching two contigs together. Additional sequencing clones from BAC libraries confirmed the misassembly. Adding the discovered SV contigs to the reference genome could rescue 2.62% of unmapped Illumina short reads, and 1.24% of the SV-contig-mapped reads show increased mapping quality, thus improving variant detection. This effect is most pronounced for insertions, for which 25.68% of the reads show increased mapping quality when compared to the reference genome. Furthermore, GATK was able to identify a substantial amount of variation within SV insertions (i.e., 68,656 alternative alleles across the 30 whole-genome haplotypes) where no reference sequence previously existed. Taken together, these data proved to be useful in re-constructing a more precise canonical human reference genome.

### Concluding remarks

Audano et al. [2] provided a sequence-resolved SV callset from analysis of 15 human genomes. They found the reported SVs to be significantly enriched with VNTRs and STRs and correlated with DSB. In addition, they found that certain SVs impact gene regulatory elements and affect gene expression, opening a door for additional future studies correlating SVs with gene expression. They further patched errors and biases in the current human reference genome assembly using their SV callset, significantly improving the quality of future short-read alignments and variant calling. This study also promotes the concept of a pan-genome (Fig. 1d), which incorporates SVs into the reference genome and can be applied to recently published graph genome tools [8, 9]. The next steps will involve phasing human genomes to reduce false negatives [10] and discovering complex SVs and indels that map to large repetitive regions of the human genome.

## Abbreviations

DSB: Double strand break
SMRT: Single-molecule, real-time
STR: Short tandem repeat
SV: Structural variant
UTR: Untranslated region
VNTR: Variable number of tandem repeats
